# Sexual health, risky sexual behavior and condom use among adolescents young adults and older adults in Chiang Mai, Thailand: findings from a population based survey

**DOI:** 10.1186/s13104-017-3055-1

**Published:** 2017-12-04

**Authors:** Kanokporn Pinyopornpanish, Sanhapan Thanamee, Wichuda Jiraporncharoen, Kanittha Thaikla, Jessica McDonald, Apinun Aramrattana, Chaisiri Angkurawaranon

**Affiliations:** 1 Department of Family Medicine, Faculty of Medicine, Chiang Mai University, Chiang Mai, 50200 Thailand; 2 Research Institute for Health Sciences, Chiang Mai University, Chiang Mai, 50200 Thailand; 3 Thai Health Promotion Foundation, Bangkok, 10120 Thailand

## Abstract

**Background:**

Sexual health is one of the key dimensions of health across all ages. Understanding risky sexual behaviors remains an important area of public health research. This study aimed to explore sexual health, risky sexual behaviors and factors associated with recent condom use as condom use is considered a main intervention proven to reduce negative health consequences of risky sexual behaviors, specifically related to sexually transmitted infections and unintended pregnancies. A stratified two-stage cluster sampling technique survey was conducted in Chiang Mai, Thailand. Information was obtained about age of first sexual intercourse, sexual activity, condom use, number of partners and history of drug/alcohol use prior to sexual activities within the past 3 months. A weighted analysis was performed to account for data clustering.

**Results:**

It is estimated that most men (93%) and women (86%) in Chiang Mai have engaged in sexual intercourse. More than 70% of the people in Chiang Mai over age 30 remained sexually active in the past 3 months, even for populations over age 50. Eight percent of male teenagers reported having more than one sexual partner in the past 3 months. Regular condom use was reported in less than 5% of the population (6.6% men and 3.1% women).

**Conclusions:**

Our study demonstrated that sexual health is an important public health issue across all age groups. Condom use has been promoted as one way to minimize and prevent unintended consequences of sexual behavior but overall use remains low.

**Electronic supplementary material:**

The online version of this article (10.1186/s13104-017-3055-1) contains supplementary material, which is available to authorized users.

## Background

Sexual health is one of the key dimensions of health across all ages. Understanding risky sexual behaviors remains an important area of public health research [1]. Risky sexual behaviors can lead to many negative health related consequences and public health issues, especially sexually transmitted infections (STIs) and unintended pregnancies [2]. High risk sexual behaviors include younger age at first sexual intercourse (SI), having multiple partners or casual partners, inappropriate use of contraception, and drug and alcohol use before sexual intercourse [3–6].

Unsafe sex was found to be one of the leading risk factors related to the global burden of disease over the past decade [7]. Many global and local public health policies promote sexual education and encourage safe sexual practices instead of prohibiting them. However, unintended pregnancy and STIs are still ongoing problems. While more effective methods are used to prevent pregnancy, STIs can be effectively prevented with male condom. Higher incidence of STIs than in the past is reported in the United States and the United Kingdom, not only in adolescents, but also in middle-aged adults and elderly populations [8, 9]. Thus, sexual health and high-risk sexual behavior is an important public health issue across all age groups.

Sexual behavior is affected by cultural values and public health policies that are different in each country and setting. Furthermore, cultural values and health policies have changed over time [10]. As a result, the sexual norms in most parts of the world are experiencing cultural transitions, including Thailand. It has been reported that while Thai people are aware of the possible transmission of human immunodeficiency virus (HIV) through sexual intercourse, risky sexual behavior are continually reported [11]. These risky behaviors include younger age at first intercourse, multiple partners, and low rate of condom use with casual partners. Data from 2012 suggested that STIs were increasing in many age groups in Thailand [12].

This study aimed to assess sexual health and risk behaviors among individuals across different age groups among those living in Chiang Mai, which has the second highest prevalence in STIs in Thailand [12] Prior studies conducted in Thailand focused mostly on adolescents [3, 11, 13–15] while a very limited amount of research has been conducted among other age groups [10, 16]. In addition, the study aimed to explore factors associated with recent condom use as condoms are considered a main intervention intended to reduce negative health consequences associated with risky sexual behaviors, specifically related to STIs [17, 18].

## Methods

### Study setting and design

This population-based survey was conducted in Chiang Mai province, the largest province in the Northern region of Thailand, in 2014. The targeted populations were those who have lived in Chiang Mai for at least 3 months between ages 15 and 64. Data was collected by trained field researchers using face-to-face structured interviews and questionnaires that were developed from questionnaires used in Thailand National Health Examination Survey [19] and the Thai National Sexual Behavior Survey [20].

### Sampling and sample size calculations

The Thai census estimated that there were 713,053 households and approximately 1.6 million people living in Chiang Mai in 2014. Approximately 1.2 million people were between 15 and 64 years of age. A stratified two-stage cluster sampling technique was done. The enumeration areas (EAs) were sub-districts in Chiang Mai and were considered the primary sampling units. Households were the secondary sampling units. Thirty-six EAs were randomly selected using probability proportional to the size of the population in each unit. Twenty-four EAs were in urban areas and 12 were in rural areas. Twenty households per cluster were randomly selected in the second stage. Allowing for an 80% response rate and a design effect of 1.5, it was estimated that 1888 people could represent the targeted population.

### Measurements and variable definitions

In addition to general demographic data, the survey obtained information on age at first sexual intercourse, sexual activity and number of partners within the past 3 months along with history of drug or alcohol use before sexual intercourse in the past 3 months. To assess condom use, participants were asked, “In the past 3 months, how often do you use (male) condoms to prevent STIs”? Participants could choose from four choices: never, sometimes, most of the time and all the time. Those who answered the latter two options were considered regular condom users. For the study, other high risk behaviors were defined as, having more than one partner (multiple partners) within the past 3 months and using drugs or alcohol prior to SI within the past 3 months.

Other factors of interests in the study survey included highest education, which was classified into four categories: primary school, early secondary school, late secondary school and Bachelors degree. Monthly income was categorized into four categories. Marital status was categorized into three categories: single, married/partner or separated/divorced/widowed. Urbanicity of living location was also documented as urban or rural.

### Analysis plan

Descriptive statistics were used to describe the sampled population in terms of age and gender distribution, marital status and urban or rural dwelling locations. To infer back to the source population, weighted analysis were conducted to account for a clustering effect using the *survey* commands in STATA 13. Prevalence of risky sexual behaviors were estimated and stratified by age and sex. Univariable and multivariable logistic regression were used to explore factors associated with regular condom use among those who have been sexually active in the past 3 months. Age and sex were considered a priori when identifying confounders in multivariable analysis.

### Ethics

Participants were given an informed consent form prior to each interview. If participants were less than 18 years of age, consent was obtained from legal guardian(s). The study was approved by an Ethics Committee from Chiang Mai University (No 62/2014).

## Results

A total of 1744 participants were enrolled in the present study and a 92.4% response rate was obtained (92.8% response in urban areas and 91.7% response in rural areas). The sample represented the source population well in terms of age and sex distributions (Additional file 1: Table S1). The sample consisted of 936 women and 808 men. The mean age of the study sample was 43.3 for women and 43.9 for men, although, about 40% were over 50 years of age. About half were educated beyond primary school (52.5%). Most were partnered (70.7%) and living in urban areas (66.1%). Approximately 70–80% of the population over the aged of 30 were married or partnered (Additional file 1: Table S2). The weighted estimates of demographic characteristics in the Chiang Mai population were similar to that of the sample (Table 1).

**Table 1:** Demographics of study sample and study population

	Female N = 936		Male N = 808		Total N = 1744	
	UW	W	UW	W	UW	W
Mean age	43.3	43.3	43.9	43.7	43.6	45.5
Age group						
15–19	6.2	6.1	4.3	4.8	5.3	5.6
20–29	14.3	14.1	15.8	16.5	15.0	15.2
30–39	14.6	14.6	17.1	16.6	15.8	15.5
40–49	24.3	24.4	20.1	19.2	22.3	22.0
50–59	29.5	29.8	29.1	29.2	29.3	29.5
Over 60	11.1	11.0	13.6	13.6	12.3	12.2
Highest education						
Primary school	51.1	51.3	43.3	42.5	47.5	47.2
Early secondary school	10.4	10.3	13.9	14.5	12.0	12.3
Late secondary school	20.6	20.1	26.5	26.3	23.3	23.1
Bachelor’s degree	17.9	18.2	16.3	16.3	17.2	17.4
Status						
Single	18.3	18.3	20.8	21.8	19.4	19.9
Married/partnered	68.9	68.0	73.9	73.1	70.7	70.4
Separated/divorced/widowed	13.8	13.7	5.3	5.1	9.9	9.7
Monthly personal income (baht)						
≤ 5000	43.5	43.0	30.5	29.4	37.2	36.4
> 5000–10,000	33.2	33.2	41.6	41.7	37.2	37.3
> 10,000–15,000	10.0	10.0	13.5	14.1	11.7	12.0
> 15,000	13.3	13.7	14.4	14.7	13.8	14.2
Location						
Rural	33.6	32.8	34.4	33.2	33.9	33.0
Urban	66.4	67.2	65.6	66.8	66.1	67.0

*UW* unweighted results, *W* weight results, *1 US dollar* approximately 35 baht in 2014

### Sexual history

It was estimated that the majority of men (92.7%) and women (86.0%) in Chiang Mai have engaged in sexual intercourse (SI). Stratified by current age group, nearly 30% of male teenagers and 20% of female teenagers have engaged in SI. By age 30, almost all (95%) participants reported engaging in previous SI (Table 2). The estimated mean ages of first SI amongst those with a history of SI were 20.5 and 20.9 years of age for men and women, respectively.

**Table 2:** Sexual history by age group

	Age group	Female	Male	Total
Mean age at first SI^b^ (95% CI)	–	20.9 (20.6–21.2)	20.5 (20.1–20.8)	20.7 (20.5–20.9)
Percent (%) who ever had SI in their lifetime (95% CI)	15–19	19.7 (11.0–32.8)	30.1 (16.3–48.8)	24.0 (15.8–34.7)
	20–29	61.5 (52.5–69.8)	83.7 (75.3–89.6)	72.8 (66.6–78.2)
	30–39	91.8 (84.9–95.7)	97.9 (75.3–89.6)	94.8 (91.0–97.1)
	40–49	96.7 (93.5–98.3)	97.9 (94.4–99.2)	97.2 (95.1–98.4)
	50–59	95.4 (92.2–97.3)	99.1 (96.3–99.8)	97.1 (95.2–98.2)
	Over 60	97.6 (92.6–99.2)	98.3 (93.3–99.6)	97.9 (95.1–99.1)
	All	86.0 (83.6–88.2)	92.7 (90.5–94.4)	89.1 (87.5–90.6)
Percent (%) had SI in the past 3 months (95% CI)	15–19	15.0 (7.8–26.7)	10.7 (4.0–25.7)	13.2 (7.7–21.7)
	20–29	45.9 (37.4–54.7)	55.9 (46.8–64.7)	51.0 (44.7–57.3)
	30–39	77.1 (68.8–83.7)	75.0 (66.6–81.9)	76.0 (70.3–81.0)
	40–49	79.4 (73.4–84.4)	82.4 (75.5–87.7)	80.6 (76.2–84.4)
	50–59	63.0 (56.9–68.8)	82.7 (76.9–87.3)	72.1 (67.9–76.0)
	Over 60	39.9 (30.7–49.9)	65.6 (55.6–74.3)	52.3 (46.3–60.1)
	All	61.2 (57.9–64.4)	71.1 (67.7–74.3)	65.8 (63.5–68.2)
Percent (%) who regularly used condoms in the past 3 months^a, b^ (95% CI)	15–19	17.2 (4.0–50.5)	23.7 (5.6–61.9)	20.5 (7.4–45.3)
	20–29	4.8 (2.0–11.3)	18.5 (11.9–27.5)	12.8 (8.6–18.7)
	30–39	7.0 (3.4–13.8)	7.1 (3.5–13.6)	7.1 (4.3–11.3)
	40–49	2.1 (0.9–5.0)	3.5 (1.6–7.7)	2.7 (1.5–4.8)
	50–59	2.2 (0.9–5.4)	3.4 (1.6–7.1)	2.8 (1.5–4.9)
	Over 60	No observations	2.8 (1.0–7.3)	1.4 (0.5–3.8)
	All	3.1 (2.1–4.7)	6.6 (4.9–8.7)	4.8 (3.7–6.1)

*SI* sexual intercourse, *CI* confidence interval

^a^Regular condom used defined as having used condom most of the time and all of the time in past 3 months

^b^The data analyzed amongst those who had experienced SI

Overall, nearly two-thirds of the population in Chiang Mai have engaged in sexual intercourse over the past 3 months. Stratified by age group, approximately 13% of teenagers and 51% of adults between ages 20 and 29 had SI in the past 3 months. This prevalence increased after age 30. Over 75% of men between 30 and 59 years old and more than 65% of men 60 and over were sexually active. While women reported having less recent sexual encounters than men, the trend in prevalence remained similar to men such that over 75% of women in their thirties and forties reported recent sexual intercourse. This number dropped to 63% among women in their fifties and approximately 40% among those age 60 and over (Table 2).

### Condom use

While recent sexual activity was common, regular condom use within the past 3 months was scarce, at less than 5% for the population. Men reported regular condom use more often than women, at 6.6 and 3.1% respectively. Stratified by age groups, higher prevalence of regular condom use was reported among male and female teenagers and early adult males, at approximately 20% (Table 2). Among those who reported prior SI, nearly 90% of women over 19 years of age reported that their partner never used a condom in the past 3 months while over 90% of men age 30 and older also reported never using condoms over the past 3 months (Table 3).

**Table 3:** Sexual risk behavior by key demographics among those with history of having had sexual intercourse (SI)

	Age group	Female	Male	Total
Percent (%) who never used condoms during SI in the past 3 months (95% CI)	15–19	63.3 (32.6–86.0)	68.0 (33.0–90.2)	65.7 (42.2–83.5)
	20–29	92.1 (84.7–96.1)	71.8 (61.9–79.9)	80.2 (73.5–85.5)
	30–39	88.7 (81.0–93.6)	90.1 (83.3–94.3)	89.4 (84.6–92.8)
	40–49	95.2 (90.8–97.6)	92.8 (87.2–96.1)	94.2 (91.1–96.3)
	50–59	97.8 (94.5–99.1)	93.9 (89.6–96.5)	95.9 (93.5–97.5)
	Over 60	100	96.4 (91.6–98.5)	98.1 (95.5–99.2)
	All	94.9 (92.9–96.3)	89.7 (87.1–91.8)	92.3 (90.8–93.7)
Proportion (per 1000) with more than 1 sexual partner in past 3 months (95% CI)	15–19	No observations	80.1 (10.5–414.3)	41.2 (5.8–244.6)
	20–29	No observations	15.9 (3.9–61.9)	9.3 (2.3–36.6)
	30–39	No observations	15.3 (3.5–63.4)	7.9 (1.8–33.3)
	40–49	No observations	6.2 (0.9–42.9)	2.6 (0.4–17.9)
	50–59	7.0 (1.7–27.9)	No observations	3.7 (0.9–14.8)
	Over 60	No observations	No observations	No observations
	All	2.3 (0.6–9.3)	7.5 (3.3–17.1)	4.8 (2.4–9.8)
Percent (%) who used alcohol/drugs before SI in past 3 months (95% CI)	15–19	No observations	No observations	No observations
	20–29	1.0 (0.1–7.0)	18.9 (12.0–28.4)	11.4 (7.3–17.6)
	30–39	1.5 (0.4–6.0)	16.3 (10.7–24.1)	9.1 (6.0–13.6)
	40–49	1.4 (0.4–4.4)	12.0 (7.5–18.7)	5.8 (3.7–8.9)
	50–59	1.3 (0.4–4.0)	11.0 (7.4–16.1)	5.9 (4.0–8.5)
	Over 60	1.0 (0.1–6.8)	2.6 (0.8–7.9)	1.8 (0.7–4.9)
	All	1.3 (0.7–2.4)	11.9 (9.6–14.7)	6.4 (5.3–7.9)

*CI* confidence interval

### Other risky sexual behaviors

Among those who have engaged in SI, 8% of male teenagers reported having more than one sexual partner in the past 3 months, while approximately 1.5% of men in their twenties and thirties reported having more than one sexual partner in the past 3 months. Among women, having multiple partners in the past 3 months was rare. Overall, it is estimated that 0.7% of men and 0.2% of women who reported a history of sexual intercourse had more than one sexual partner in the past 3 months (Table 3). Within the past 3 months, around 1–1.5% of women across all age groups reported using drugs or alcohol before SI. However, the prevalence is over 10 times higher among men. Up to 20% of male teenagers reported using drugs or alcohol prior to sexual intercourse (Table 3).

### Factor associated with regular condom use

Among those who were sexually active in the past 3 months, there were many factors associated with regular condom use. In a crude analysis, increasing age was associated with declining condom use. Males reported more condom use compared to females. Respondents without a partner were more likely to use condoms than those who identified as attached. Higher education and higher income were associated with condom use. People with multiple partners or drug use before having SI were also more likely to report regular condom use over the past 3 months (Table 4).

**Table 4:** Factors associated with regular condom use during sexual intercourse (SI) in past 3 months

	Crude odds ratio (OR)	Adjusted OR	p value
Age group in years			0.31*
15–19	Reference	Reference	
20–29	0.77 (0.19–3.11)	2.93 (0.63–13.7)	
30–39	0.29 (0.07–1.24)	2.20 (0.53–9.08)	
40–49	0.10 (0.02–0.45)	1.12 (0.26–4.84)	
50–59	0.12 (0.28–0.53)	1.45 (0.35–5.96)	
Over 60	0.07 (0.01–0.40)	–	
Age at first SI (increase)	0.98 (0.89–1.06)	1.06 (0.97–1.15)	0.17
Sex (male)	1.84 (1.07–3.16)	1.34 (0.72–2.49)	0.35
Marital status			< 0.01*
Widowed/separated/divorced	Reference	Reference	
Married/partnered	1.16 (0.15–8.88)	1.23 (0.14–10.8)	
Single	18.5 (2.31–147.9)	9.49 (1.02–88.7)	
Monthly personal (increase)	1.88 (1.50–2.36)	1.87 (1.31–2.65)	< 0.01
Location			
Urban	Reference	Reference	
Rural	1.41 (0.81–2.43)	0.71 (0.35–1.42)	0.33
Highest education (increase)	1.84 (1.49–2.28)	1.06 (0.75–1.50)	0.74
More than 1 partner in past 3 months	34.0 (7.77–149.2)	41.6 (6.59–262.7)	< 0.01
Use alcohol/drugs before SI in past 3 months	2.42 (1.13–5.17)	1.02 (0.43–2.46)	0.96

Analysis conducted among those who had a history of having had sexual intercourse in the past 3 months. Regular condom used defined as having used condom most of the time and all of the time in past 3 months: * overall p value using likelihood ratio test

The direction of associations in an adjusted analysis was similar to those of the crude analysis. In the multivariable analysis, results showed that being single and having an above average income was associated with regular condom use. Regular condom use was more commonly reported among those with multiple partners and those who used drugs or alcohol prior to SI. For age, the adjusted analysis found that teenagers were less likely to use condoms compared to older age groups (Table 4).

## Discussion

This study indicates that more emphasis should be placed on sexual health in Chiang Mai as the majority of study participants reported past sexual activity. Moreover, it is estimated that over half of the population in Chiang Mai, over age 20, reported having SI in the past 3 months. In addition, many of those who engaged in SI reported low rates of condom use. On a global scale, condom use is promoted as a primary method to prevent undesirable health outcomes, specifically sexually transmitted infections [21, 22]. However, low rates of condom use were found among the study population, which enhances the need for further investigation into a lack of condom use in Chiang Mai, Thailand.

The average age at first SI reported by the study population was approximately 20 years of age. This finding is similar to results from the 2006 Thai National Survey of Sexual Behavior which revealed the average age at first SI was 19 for men and 20 for women [10]. With a cumulative effect, it was not surprising that increasing age was associated with increasing percentage of ever having SI throughout lifetime. Moreover, 90% of the population over age 30 had already engaged in SI. This finding supports that sex education should be provided during teenage years prior to the onset of sexual activity.

In line with the previous study from Thailand by Ford and Chamratrithirong [10], our study shows declining prevalence of recent sexual activity in aging people. This may be due to their general health conditions, emotional desire, partner characteristics and family context [16, 23]. However, our data demonstrates that the majority of the people living in Chiang Mai, over age 30, still remain sexually active, even for populations over age 50. The proportion who remain sexually active in older populations were similar to a US study, which found that 73% of participants aged 57–64 years were still sexually active [23]. This information supports that health education on safe sexual practices and family planning for men and women who are of reproductive age should be continuously promoted throughout their lifetime.

In Thailand, many public health campaigns exist to promote condom use. In 1989, Thailand launched the 100% condom campaign, which targeted sex workers and their clients [24]. Later campaigns have targeted condom use for the general population, heavily targeting teenagers in order to prevent teenage pregnancies [25]. However, our research indicates low levels of condom use in Chiang Mai across all age groups. This was similar to results from the 2006 Thai National Survey which found that approximately 6.6% of Thai men routinely use condoms with their regular partners [20].

In particular, our study demonstrated that older age groups, between 30 and 50 years of age, remain sexually active but also had low levels of condom use. It is possible that they are more likely to be married and/or have regular partners thus resulting in lower regular condom use or no condom use. However, there is still a risk of transmitting STIs to regular partners if prior exposure was a factor with previous partners. A previous survey found that among older Thai nationals, one quarter married their first sexual partner and the majority of Thai men had casual partners or more than one partner in their lifetime [10]. While currently less common, in previous decades, high levels of extramarital sex has been reported particularly with sex workers [26]. Our data also suggest that while the majority of men over the aged of 30 are married or partnered, approximately 1% have had more than one sexual partner in the past 3 months.

In the present study, men were more likely than women to report condom use. A previous study from Northern Thailand by Tangmunkongvorakul [11] found that men were more likely to have non-regular sexual partners and likely to report more condom use, in order to prevent STIs from those casual partners. The study also reported that some women were unable to negotiate protected sex with their partner because they respected the man’s decision or had prior uncomfortable experiences after asking their partner to use condom. This finding could reflect Thai cultural values in that men have authority and women appear less concerned about their own risks and are more likely to accept sexual risk factors to avoid negative feedback from their partner [27]. Higher condom use rates are associated with higher education in our study and in previous studies [20, 28]. In Thailand as sex education is offered in school, those with higher education could be more aware of reproductive health issues [29]. However, increasing sexual education to general population across age groups, especially in reproductive women, could help reduce the risk of STIs and unwanted pregnancies [30]. Innovative strategies driven by non-government organizations such as peer youth education programs may be useful [31].

We found that those involved in other risky behaviors, such as, SI with multiple partners and drug/alcohol use prior to SI, were more likely to report condom use than those who did not report risky sexual behaviors. Another study reported that alcohol was associated with an increased risk of engaging in sexual activities and an increased number of multiple or casual partners [32]. Our findings could potentially reflect respondent awareness about high risk sexual behaviors but this issue needs to be further investigated. However, as the overall prevalence of regular condom use among high risk groups remains low, promotion and prevention policies targeting this group are still needed.

The present study highlights other factors that may be associated with condom use, specifically showing no significant difference in condom use between rural and urban populations. However, differences in personal income were significantly associated with condom use. Personal income was a major confounding factor in the association between age and condom use. This reflects that condom price could be one of the key barriers to regular condom use in Thailand as seen in other developing countries [33]. The Thai policy for access to free condoms [24, 34] should be expanded and highlighted to improve accessibility to condom and increase the use in Thailand.

A major strength of the present study was proper sampling and analysis, which is representative to the targeted population. Population surveys are useful in reflecting the current situation and to help guide future public health interventions and preventive strategies. There were also a number of limitations, including the cross-sectional design limit causal interpretation. Our categorization of regular condom use included those who reported using condoms most of the time. Furthermore, we could not assess proper use of condoms such as whether there were no slippages or no early removals. These limitations will effect the implications of our findings as it may not result in effective prevention of STIs. Due to the nature of the survey, difficult to access and vulnerable populations for high-risk sexual behaviors such as sex workers, injected drug users and high risk youths are unlikely to be captured in the survey and thus likely to be under-represented in the present study. Face-to-face interviews may have also generated some response bias. Both aforementioned issues were likely to result in an underestimation of high-risk sexual behaviors. Further subgroup analysis were not possible as the study did not collect detailed data on type of sexual partner (regular or casual), sexual orientation and personal history of STIs. Due to limited sample size, we could not assess condom use amongst those with more than one partner within the past 3 months. Further studies are needed to explore the detailed barriers and perceptions of condom use among different ages, gender and socioeconomic status in order to guide effective condom policies in Thailand.

## Conclusions

In conclusion, our study demonstrated that sexual health and risky sexual behavior is an important public health issue across all age groups. Condom use has been promoted as one way to minimize and prevent unintended consequences of sexual behavior but overall use remains low even among high-risk populations in Chiang Mai, Thailand. Our study suggests that condom price may be a barrier to condom use and that sex education in Thailand should focus on the risks of unprotected sexual activity and tackle social, gender and cultural perceptions that may inhibit condom use. Thailand recently launched its new National Condom Strategy for 2015–2019 [35]. Some of the strategies are aimed to tackle such issues by providing free condoms during Valentine’s day or cheap condoms in public events and in public areas such as department store and hotel restrooms. The National Condom Strategy also includes mass media campaigns to promote safe sex and positive perceptions about condom use. Evaluation is still needed to estimate the effectiveness of such policies moving forward.

## Additional file

**Additional file 1: Table S1.** Characteristics of sampled population and source population. **Table S2.** Relationship status by age group and sex.

## Abbreviations

EAs: enumeration areas
HIV: human immunodeficiency virus
SI: sexual intercourse
STIs: sexually transmitted infections
