# Supplementary material for: Sexual health, risky sexual behavior and condom use among adolescents young adults and older adults in Chiang Mai, Thailand: findings from a population based survey
Source: BMC Res Notes. 2017 Dec 4;10:682. doi: 10.1186/s13104-017-3055-1 (PMC5715516; doi:10.1186/s13104-017-3055-1)
Supplement: Supplementary file 1 — Additional file 1: Table S1. Characteristics of sampled population and source population. Table S2. Relationship status by age group and sex. [file 13104_2017_3055_MOESM1_ESM.docx]

Table S1 Characteristics of sampled population and source population

|  | Age Group | | | | | | Total |
| --- | --- | --- | --- | --- | --- | --- | --- |
|  | 15-19 | 20-29 | 30-39 | 40-49 | 50-59 | 60+ |  |
| Men |  |  |  |  |  |  |  |
| Total number of men Chiang Mai | 29897 | 102225 | 102608 | 118944 | 180816 | 84026 | 618516 |
| Proportion of men in Chiang Mai | 41.1 | 50.8 | 49.9 | 40.9 | 46.3 | 52.1 | 46.8 |
| Total number of men in sample | 35 | 128 | 138 | 162 | 235 | 110 | 808 |
| Proportion of men in sample | 37.6 | 48.9 | 50.2 | 41.6 | 46.0 | 51.4 | 46.3 |
| Estimated proportion of men in Chiang Mai (weighted analysis) | 41.1 | 50.8 | 50.0 | 40.9 | 46.3 | 52.1 | 46.8 |
| Women |  |  |  |  |  |  |  |
| Total number of women Chiang Mai | 42920 | 99124 | 102836 | 171982 | 209821 | 77217 | 703900 |
| Proportion of women in Chiang Mai | 58.9 | 49.2 | 50.1 | 59.1 | 53.7 | 47.9 | 53.2 |
| Total number of women in sample | 58 | 134 | 137 | 227 | 276 | 104 | 936 |
| Proportion of women in sample | 62.4 | 51.1 | 49.8 | 58.4 | 54.0 | 48.6 | 53.7 |
| Estimated proportion of women in Chiang Mai (weighted analysis) | 58.9 | 49.2 | 50.0 | 59.1 | 53.7 | 47.9 | 53.2 |

Table S2: Relationship status by age group and sex

|  |  | Relationship status | | |
| --- | --- | --- | --- | --- |
|  | Age group | Single | Married/  partnered | Separated/  divorce/widowed |
| Female | 15-19 (n=58) | 95.3  (86.2 to 98.5) | 4.7  (1.5 to 13.8) | no observation |
|  | 20-29 (n=134) | 55.6  (46.7 to 64.1) | 38.1  (30.1 to 46.9) | 6.3  (3.0 to 12.5) |
|  | 30-39 (n=137) | 11.3  (7.7 to 18.4) | 77.1  (68.9 to 83.7) | 11.6  (7.1 to 18.2) |
|  | 40-49 (n=227) | 4.6  (2.3 to 8.6) | 81.4  (75.6 to 86.2) | 13.9  (9.8 to 19.4) |
|  | 50-59 (n=276) | 5.3  (3.2 to 8.9) | 79.4  (74.0 to 83.9) | 15.2  (11.4 to 20.2) |
|  | over 60 (n=104) | 2.4  (0.7 to 7.4) | 68.5  (58.8 to 86.8) | 29.1  (21.0 to 38.7) |
|  | All (n=936) | 18.3  (15.8 to 21.0) | 68.0  (64.8 to 71.0) | 13.7  (11.6 to 16.2) |
| Male | 15-19 (n=35) | 96.8  (80.2 to 99.5) | 3.2  (0.0 to 19.8) | no observation |
|  | 20-29 (n=128) | 62.8  (53.7 to 71.1) | 34.8  (26.7 to 43.8) | 2.4  (0.8 to 7.4) |
|  | 30-39 (n=138) | 24.9  (17.7 to 33.5) | 68.9  (60.0 to 76.5) | 6.3  (3.2 to 11.9) |
|  | 40-49 (n=162) | 8.4  (4.9 to 14.0) | 85.7  (79.0 to 90.5) | 5.9  (3.0 to 11.4) |
|  | 50-59 (n=235) | 2.2  (0.9 to 5.2) | 93.1  (89.2 to 95.7) | 4.7  (2.7 to 8.1) |
|  | over 60 (n=110) | 2.9  (1.1 to 7.7) | 88.6  (80.6 to 93.5) | 8.5  (4.3 to 16.2) |
|  | All (n=808) | 21.8  (18.6 to 25.0) | 73.1  (69.7 to 76.2) | 5.1  (3.7 to 6.9) |

Results represents row percentage; CI=confidence interval
